# Aging Predisposes B cells to Malignancy by Activating c-Myc and Perturbing the Genome and Epigenome

**DOI:** 10.1093/geroni/igab046.2152

**Published:** 2021-12-17

**Authors:** Anastasia Shindyapina, Jose Castro, Alessandro Barbieri, João Paulo, Olga Strelkova, John Sedivy, John Manis, Vadim Gladyshev

**Affiliations:** 1 Brigham and Women's Hospital, Harvard Medical School, Massachusetts, United States; 2 I 3.S, Porto, Porto, Portugal; 3 Boston Children's Hospital, Boston, Massachusetts, United States; 4 Harvard Medical School, Boston, Massachusetts, United States; 5 Brown University, Providence, Rhode Island, United States; 6 Brigham and Women's Hospital, Boston, Massachusetts, United States

## Abstract

Age is the single major risk factor for human cancer, but naturally occurring cancers are rarely studied in aging models. Like humans, mice spontaneously develop cancer with age, and standard laboratory strains are predisposed for B-cell lymphoma. Here, we uncover how B-cell lymphoma develops as a consequence of the aging immune system. We found that aged B cells acquire somatic mutations in tumor suppressors and oncogenes (e.g. Trp53, Pim1, and Myh11) and undergo monoclonal expansions, with some clones representing 86% of splenic B cells. Clonal B cells had hypermethylated promoters and globally silenced expression, suggesting a role of DNA methylation in clonal selection of premalignant B cells. B-cell size, spleen weight, and a novel population of B cells, which we named Myc+ cells, emerged as convenient markers of malignancy. High-throughput analyses of clonal B cells and the use of genetic mouse models revealed that c-Myc drives B-cell size increase and clonal expansion with age. Phoshoproteome and co-culture experiments revealed that c-Myc is activated by signals from the aging microenvironment. Moreover, single-cell RNA-seq suggested that clonal B cells originate from age-associated B cells, further underlying the importance of aging environment in cancer transformation. Longitudinal analyses demonstrated a negative impact of premalignant B cells on mouse lifespan and linked it to age-related myeloid bias. Together, our study revealed cell-autonomous changes that cooperate with the aging microenvironment to give rise to preneoplastic B cells. This stidy established a novel model to study how aging predisposes cells to cancer transformation.

